# Response and recovery mechanisms of river microorganisms to gradient concentrations of estrogen

**DOI:** 10.3389/fmicb.2023.1109311

**Published:** 2023-02-08

**Authors:** Dan Qin, Yan Li, Nengwang Chen, Anyi Hu, Chang-Ping Yu

**Affiliations:** ^1^CAS Key Laboratory of Urban Pollutant Conversion, Fujian Key Laboratory of Watershed Ecology, Institute of Urban Environment, Chinese Academy of Sciences, Xiamen, China; ^2^School of Ecological Environment and Urban Construction, Fujian University of Technology, Fuzhou, Fujian, China; ^3^Fujian Provincial Key Laboratory for Coastal Ecology and Environmental Studies, College of the Environment and Ecology, Xiamen University, Xiamen, China

**Keywords:** estrogen, bioremediation, gradient concentration, microbial diversity, assembly mechanism

## Abstract

As an important ecological system on the earth, rivers have been influenced by the rapid development of urbanization, industrialization, and anthropogenic activities. Increasingly more emerging contaminants, such as estrogens, are discharged into the river environment. In this study, we conducted river water microcosmic experiments using *in situ* water to investigate the response mechanisms of microbial community when exposed to different concentrations of target estrogen (estrone, E1). Results showed that both exposure time and concentrations shaped the diversity of microbial community when exposed to E1. Deterministic process played a vital role in influencing microbial community over the entire sampling period. The influence of E1 on microbial community could last for a longer time even after the E1 has been degraded. The microbial community structure could not be restored to the undisturbed state by E1, even if disturbed by low concentrations of E1(1 μg/L and 10 μg/L) for a short time. Our study suggests that estrogens could cause long-term disturbance to the microbial community of river water ecosystem and provides a theoretical basis for assessing the environmental risk of estrogens in rivers.

## Introduction

Freshwater accounts for only 3% of the world’s water resources, even though it feeds almost the entire population. It is estimated that over 1 billion people do not have adequate potable water, and the number will increase to 2.5 billion in 2025 (Komesli et al., 2017). Rivers serve as important sources of freshwater and have been influenced by the rapid development of urbanization and industrialization and anthropogenic activities (Yuan et al., 2022), such as traffic emissions, agricultural intensification, industrial development and aquaculture (Zeglin, 2015). These anthropogenic activities accelerate the discharge of environmental pollutants into aquatic ecosystems (Fang et al., 2022). Rivers are considered one of the most vulnerable ecosystems in the context of population growth and increased anthropogenic pressures along river bodies (Hu et al., 2017). Increasingly pollutants are detected in rivers worldwide (Vorosmarty et al., 2010). These pollutants include heavy metals (Wang et al., 2021), persistent organic pollutants (Lee et al., 2022), pharmaceutical and personal care products (PPCPs) (Peng et al., 2017), endocrine disrupting chemicals (EDCs) (Yao et al., 2018), antibiotic resistance genes (Calero-Caceres et al., 2017) and other emerging contaminants from domestic sewage, industrial effluent and agricultural runoff (Lee et al., 2022). Even so, rivers play significant roles in the sustainable development of a city. They are critical sources of water for drinking (Vorosmarty et al., 2010), agriculture, and industrial production, as well as transportation channels, pollution purification sites, and valuable reservoirs of biodiversity. They also connect terrestrial and marine ecosystems (Dudgeon et al., 2006; Xu et al., 2022).

Among all the pollutants, endocrine disruptors have been of concern because of their endocrine-disrupting effects on aquatic organisms at very low concentrations (Kidd et al., 2007; Rose et al., 2013). Especially, estrogens are identified as a first-class carcinogen by the World Health Organization due to their carcinogenic, teratogenic, and mutagenic effects (Chen et al., 2017). Owing to its environmental hazards, researchers have been studying the removal methods of estrogen for many years, such as microbial degradation (Johnson et al., 2000; Fujii et al., 2002; Kurisu et al., 2010). However, little is known about the biotic and abiotic factors affecting estrogen migration and transformation behavior in the environment. Estrogens potentially influence the diversity and function of freshwater microbial communities, which are important indexes to evaluate river quality (Hu et al., 2014; Savio et al., 2015; Meziti et al., 2016). These interference effects were assessed using several laboratory-scale microcosm or mesocosm experiments (Yergeau et al., 2012; Guasch et al., 2016). Studies have also investigated the ecological mechanisms controlling microbial community assemblies and interspecies interactions in rivers, which have been disturbed anthropogenically (Gad et al., 2020; Mainetti et al., 2021; Vilela et al., 2021). It has been suggested that estrogens and other endocrine disruptors have contrasting ecological effects on the aquatic microbiology community. To some degrading bacteria in the *in-situ* environment, estrogens become the carbon and energy sources, which is called the subsidy effect. Similar to PPCPs and other emerging contaminants, estrogens may also be toxic to other bacteria, which is called the stress effect (Luo et al., 2014). These two effects change the diversity and composition of freshwater microbial communities and relevant biogeochemical processes (Yergeau et al., 2012). While estrogen is distributed in very low environmental concentrations (ng/L–μg/L range), previous studies added high concentrations of estrogen to the environmental water samples and found that estrogen strongly stimulated *in-situ* microorganisms and enriched estrogen-degrading bacteria (Chen et al., 2018; Zhao et al., 2021). However, how low concentrations of estrogen influence the diversity of microbial communities is still unclear.

Some researchers have claimed that when environmental bacteria are exposed to some contaminants such as estrogens, the microbial community assembly mechanisms tend to be disordered, similar to the Anna Karenina principle (Ahmed et al., 2019; Zhu et al., 2022). The Anna Karenina principle is proposed for animal microbiomes (Zaneveld et al., 2017) and states that dysbiotic or stressed organisms have more variable and unstable microbiomes than healthy ones. Several findings support that this theory is suitable for environmental microbiomes. Numerous studies have demonstrated that deterministic changes in the environmental selection and biotic interaction processes are simultaneously responsible for shaping the microbial community assembly (Peng et al., 2020). However, whether the mechanisms in the assembly and changes in the microbiology community structure are similar when exposed to disparate concentrations of estrogen remains to be determined through further research (Zhang et al., 2022). Considering that low concentrations of estrogen may be degraded in a short period of time, how long will the influence of estrogens on microbial community structure remain after the estrogen has been degraded? Moreover, what are the differences between the assembly mechanisms of microbial community structures after the elimination of estrogen interference, and what are the characteristics of microbial community structures under continuous estrogen interference? To answer these questions, we sampled water from two sites in the Jiulong River and constructed three treatment groups (adding 1 μg/l, 10 μg/l, 100 μg/l estrone (E1)) and a blank group (without E1). Then, the rate of degradation of different concentrations of estrogen added to the *in-situ* water samples was studied. Meanwhile, regular samples were taken to study the changes in microbial community structure and assembly mechanisms to explore the interference effects of concentrations and time series on riverine microorganisms. This study will provide a theoretical basis for assessing the environmental risk of estrogens in river microbiome.

## Materials and methods

### Chemicals and reagents

All the solvents for liquid chromatography–tandem mass spectrometry were provided by Merck Inc. Chemical E1 was purchased from Sigma-Aldrich (United States). Oasis HLB solid phase extraction (SPE) cartridges (60 mg, 3 ml) were procured from ThermoFisher Scientific (United States). Milli-Q water purification system (Millipore, United States) provided all the water for reagents and buffers. The stock of E1 standard solution was prepared in methanol with a concentration of 1,000 mg/l and refrigerated at −20°C in the dark.

### Water sampling and gradient degradation experiment

As the second largest river in Fujian Province, China, the Jiulong River (comprising the North River and West River) has been exposed to excess nutrients for a long time (>20 years), which has led to its eutrophication (Wu G. et al., 2019). Previous investigations also showed that the Jiulong River was contaminated with multitudinous chemical micropollutants with high detection frequency, such as PPCPs (Lv et al., 2014), EDCs (Zhang et al., 2012; Ashfaq et al., 2019) and polycyclic aromatic hydrocarbons (Wu Y. et al., 2019). Compared with the West River, the North River of the Jiulong River is more affected by human production and living activities such as pig farms (Jiang et al., 2013). Therefore, it is an ideal sampling site to study the interference effect of micropollutants on microbial community structure. We sampled waters from two sites in the North River of the Jiulong River in July, 2019, labeled as N2 and N15. Information on water quality parameters, namely temperature, pH, electronic conductivity (EC), dissolved oxygen (DO), ammonium (NH_4_^+^), nitrate (NO_3_^−^), nitrite (NO_2_^−^) were determined *in situ* using YSI650 MDS meter with a multiprobe (YSI, Yellow Springs, OH, United States) and a flow injection analyzer (QC8500, Lachat VR, Loveland, CO, United States). Details of the results are provided in supplementary material. In addition to these water samples, about 12 L of water was also sampled from these two sites for the next microcosmic experiments.

After being transported to the laboratory in ice-packed coolers, the water was first filtered using 0.4 μm Sterivex GP filters (Millipore, Bedford, MA, United States) to remove contaminants, such as large particles and algae. Next, 1.5 l of the water sample was incubated in a 3-L conical flask in the dark at 25°C under the following conditions: water alone (labeled as N2 and N15); water with 1 μg/l,10 μg/l and 100 μg/l E1 (labeled as N2_1, N2_10 and N2_100; N15_1, N15_10, and N15_100, respectively). 0.5 L sterilized water from sites N2 and N15 with 1 μg/l, 10 μg/l and 100 μg/l E1 was labeled as kill control. Each treatment group was set up in triplicate. The samples (100 ml) were withdrawn from the flasks at different time (samples for E1 detection were withdrawn at 0, 12, 24, 48, 72, 96, 120, 144, 168 and 264 h; samples for DNA extraction were withdrawn at 0, 24, 72, 120, 168 and 264 h), and water samples were first filtered using 0.22-μm Sterivex GP filters. The filtered water samples with 1 μg/l and 10 μg/l E1 were pretreated using solid phase extraction (SPE) according to the EPA method 1,694 (Lv et al., 2014) and stored at −20°C in amber vials for detection of E1. The filtered water samples with 100 μg/l E1 were stored at −20°C directly without SPE. E1 analysis was performed using high performance liquid chromatography (HPLC, Shimadzu LC-20A, Japan) electrospray ion sources with triple quadrupole mass spectrometry (QqQ-MS/MS, Applied Biosystems ABI 6500, United States), with the two highest characteristic precursor ion/product ion transition pairs. Chromatographic separation was performed using a Kinetex C18 column (Acquity UPLC BEH C18, 2.6 μm, 100 × 2.1 mm; Waters) at a flow rate of 0.4 ml/min with the solvent A as 2% (v/v) acetonitrile containing 0.1% (v/v) formic acid in Milli-Q water and solvent B as methanol containing 0.1% (v/v) formic acid. The sample injection volume was 10 μl (Sun et al., 2017). Selected filters were refrigerated at −80°C until DNA extraction.

### DNA extraction and the sequencing of 16S rRNA genes

A total of 122 filters from the gradient degradation microcosmic experiments were cut into pieces with sterile scalpels, and then the DNA was extracted using the FastDNA Spinkit (Qbiogene-MP Biomedicals, Irvine, CA, United States) according to the manufacturer’s protocol (Bandopadhyay et al., 2018). The V4–V5 region of the 16S rRNA gene was amplified using the 16S rRNA universal primer 515 forward (5ʹ-GTGYCA GCM GCC GCG GTA-3ʹ) and 907 reverse (5ʹ-CCG YCA ATT YMT TTR AGTTT-3ʹ) (Gad et al., 2020). The PCR amplification was processed in a 25-μL system reacted by TransGen AP221-02 TransStart Fastpfu DNA polymerase (TransGen Biotech, China) according to the manufacturer’s protocol, and containing 2.5 μl of 10 × TransStart FastPfu buffer, 2.5 mM dNTPs, 0.4 μM primers, 0.5 μl of FastPfu polymerase, 5 μg of bovine serum albumin (Sigma, Steinheim, Germany), 20 ng of template DNA, and finally ddH_2_O up to 25 μl, with three replicates per sample. The amplification conditions were: initial denaturation at 95°C for 5 min, 25 cycles of 95°C for 30 s, 55°C for 30 s, and 72°C for 90 s, with an extension at 72°C for 10 min. Then the PCR products were purified and sequenced using the Illumina MiSeq platform (Illumina Inc., San Diego, CA, United States) with the paried-end approach (2 × 300 bp) at Majorbio Bio-Pharm Technology Co., Ltd. (Shanghai, China).

### Microbial community and statistical analysis

The raw 16S rRNA gene sequence reads were spliced and pair-ended using FLASH (1.2.11) (Magoc and Salzberg, 2011) and quality-filtered using QIIME (Quantitative Insights Into Microbial Ecology, v 1.9.1) (Fan et al., 2019). Each sample was normalized to 87,699 effective sequences at the same sequencing depth. Operational taxonomic units (OTUs) were determined based on a 97% similarity cluster using UPARSE version 7.0.1090. Chimeras were filtered using USEARCH, and the low-abundance sequences (*n* < 8) were discarded (Zhao et al., 2021). The taxonomy of each OTU was assigned using the Ribosomal Database Project classifier (version 2.2) (Hu et al., 2020) with a bootstrap cut-off of 80% (Wang et al., 2007).

The α-diversity indexes, namely Sobs, Shannon, Simpson, Ace, Chao and Coverage, were calculated using the Mothur software (1.30.2) and the normalized dataset. The Wilcoxon rank-sum test was used to evaluate the α-diversity of all the samples. The variation in the microbial community was presented as bar charts. In the β-diversity analysis, principal co-ordinates analysis (PCoA) analysis based on the Bray-Curtis (999 permutations) index with analysis of similarities (ANOSIM) was performed to evaluate the driving forces between groups. For all statistical tests, significance was confirmed at *p* < 0.05.

### Analysis of community assembly by neutral community model

The Sloan community neutral model is an adaption of the neutral theory, which is applied to evaluate the effects of stochastic processes on the assembly of prokaryotic communities (Sloan et al., 2006), containing random dispersal and ecological drift, such as mortality, migration, speciation and limited dispersal. To explain the potential importance of deterministic and stochastic processes on the community assembly when different concentrations of E1 were added to the water samples, we used the neutral community model (NCM) to predict the composition of neutrally (inside model predictions) and non-neutrally (outside model predictions) distributed OTUs by their detection frequency and relative abundance (Burns et al., 2016). The parameter R^2^ indicates the overall goodness of fit of this model. Higher R^2^ represents the community is closer to the neutral model. In other words, the community construction is more influenced by stochastic processes and less influenced by deterministic processes. Nm, the product of metamoebium size (N) and mobility (m), quantifies estimates of dispersal between communities and determines the correlation between occurrence frequency and regional relative abundance. Calculation of 95% confidence intervals around all fitting statistics was done by bootstrapping with 1,000 bootstrap replicates (Gad et al., 2020; Li et al., 2020). In this study, we separately used the datasets from different groups treated by different concentrations of E1, and OTUs from different groups were subsequently sorted into three partitions depending on whether they occurred more frequently than (‘above’ partition), less frequently than (‘below’ partition) or within (‘neutral’ partition) the 95% confidence interval of the neutral model predictions. All computations were performed in R (version 3.2.3), and the codes were downloaded from previous reference literature (Chen et al., 2019).

## Results

### Degradation of different concentrations of E1 in two water samples

**Figure 1:**
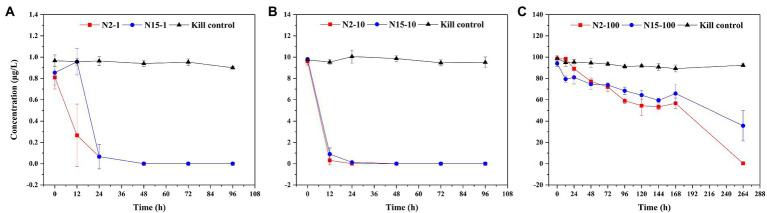
Degradation curves of E1 in sample water from sites N2 and N15 with different concentrations: **(A)** E1 with a concentration of 1 μg/l; **(B)** E1 with a concentration of 10 μg/l; **(C)** E1 with a concentration of 100 μg/l. The red square represents a sample from site N2; the blue circle represents a sample from site N15; the black triangle represents the kill control.

Throughout the microcosmic degradation experiment, all the flasks were kept in the dark conditions to ensure that the E1 was metabolized by bacteria. E1 with concentrations of 1 μg/l and 10 μg/l in both samples (N2 and N15) was quickly degraded below the detection limit (BDL). From 0 to 24 h, in the N2 samples, the concentration of E1 decreased from 0.70–0.92 μg/l to BDL −0.2 μg/l in the treatment group added 1 μg/l E1 and decreased from 9.2–9.9 μg/l to BDL μg/L in the treatment group added 10 μg/l E1; in the N15 samples, the concentration of E1 changed from 0.74–0.97 μg/l to BDL −0.2 μg/l in the treatment group added 1 μg/l E1 and decreased from 8.83–9.78 μg/l to 0.08–0.27 μg/l in the treatment group added 10 μg/l E1. However, E1 with a concentration of 100 μg/l in both samples was slowly degraded until 264 h (Figure 1). The results show that the degradation time was prolonged with the high E1 concentrations. Higher estrogen concentrations could indicate greater biotoxicity, and it may take longer time for the *in-situ* microbial community to adapt to this external stimulus under a high concentration of estrogen exposure. To further understand the stimulation of high and low estrogen concentrations on microbial community structure in the *in-situ* water samples, we also studied the changes in the microbial community based on multiple analysis methods.

### Both time and concentrations shaped the diversities of microbiota when exposed to E1

**Figure 2:**
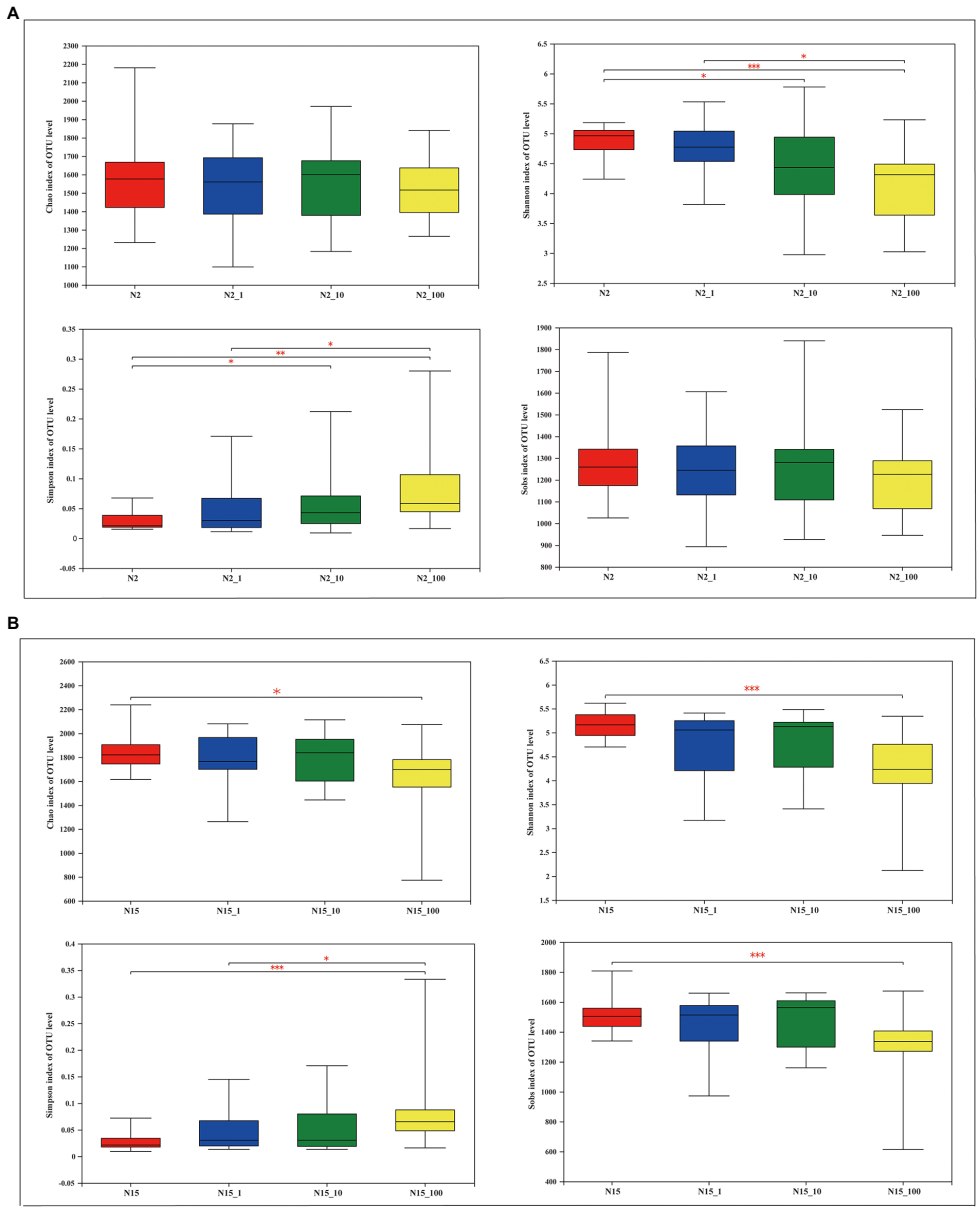
Overview of α-diversity measurements at the OTU level for different concentrations of E1 in the water samples from sites N2 and N15. **(A)** Site N2: the four images represent the Chao richness estimator, Shannon index, Simpson index and Sobs index; **(B)** Site N15: the four images represent the Chao richness estimator, Shannon index, Simpson index and Sobs index. *Indicates 0.01 < *p* ≤ 0.05, **indicates 0.001 < *p* ≤ 0.01, *** indicates *p* ≤ 0.001.

A total of 10,699,271 high-quality sequences were obtained after a quality control check among the 122 samples. The average length was 427 bp. The sequences were grouped into 13,962 OTUs at 97% identity. The results of the α-diversity estimators, namely Chao, Shannon, Simpson and Sobs indexes, showed different significance in the treatment groups with different concentrations of the two water samples (Figure 2). For instance, the Chao index was not significantly different between samples treated with different concentrations of E1 at site N2, while it was significantly different between the water samples without E1 and with 100 μg/l E1 (0.01 < *p* ≤ 0.05) at site N15. The Shannon index at N2 shows a similar significance pattern between groups without E1 and with 10 μg/l E1 and the groups treated with 1 μg/l and 100 μg/l E1 (0.01 < *p* ≤ 0.05). The *p*-value was ≤0.001 between the N2 and N2_100 groups. At site N15, there was no significant difference except in the N15 and N15_100 groups (*p* ≤ 0.001). The Simpson index at site N2 was similar between groups N2 and N2_10 and groups N2_1 and N2_100, while the *p*-values between groups N2 and N2_100 were > 0.001 and < 0.01, which was different from the pattern of the Shannon index. The Simpson index at sample N15 was only significantly different between groups N15 and N15_100 (*p* ≤ 0.001) and groups N15_1 and N15_100 (0.001 < *p* ≤ 0.01). There was no significant difference between all the treatment groups at site N2 based on the Sobs index, but it was significantly different for the groups N15 with N15_100. All the variations based on the α-diversity estimators were tested using Wilcoxon rank-sum test. Except for the Simpson index, all the other indexes decreased when the *in-situ* water samples were exposed to E1 as ambient pressure at both sites, N2 and N15, especially when exposed to 100 μg/l E1. Similarly, the OTUs from the E1 treatment also decreased with 100 μg/l E1 compared with other treatment groups with lower concentrations of E1 and without E1. The results suggested that divergent conclusions may be drawn based on different α-diversity indexes. Therefore, it is necessary to analyze all indexes when conducting a diversity comparison.

**Figure 3:**
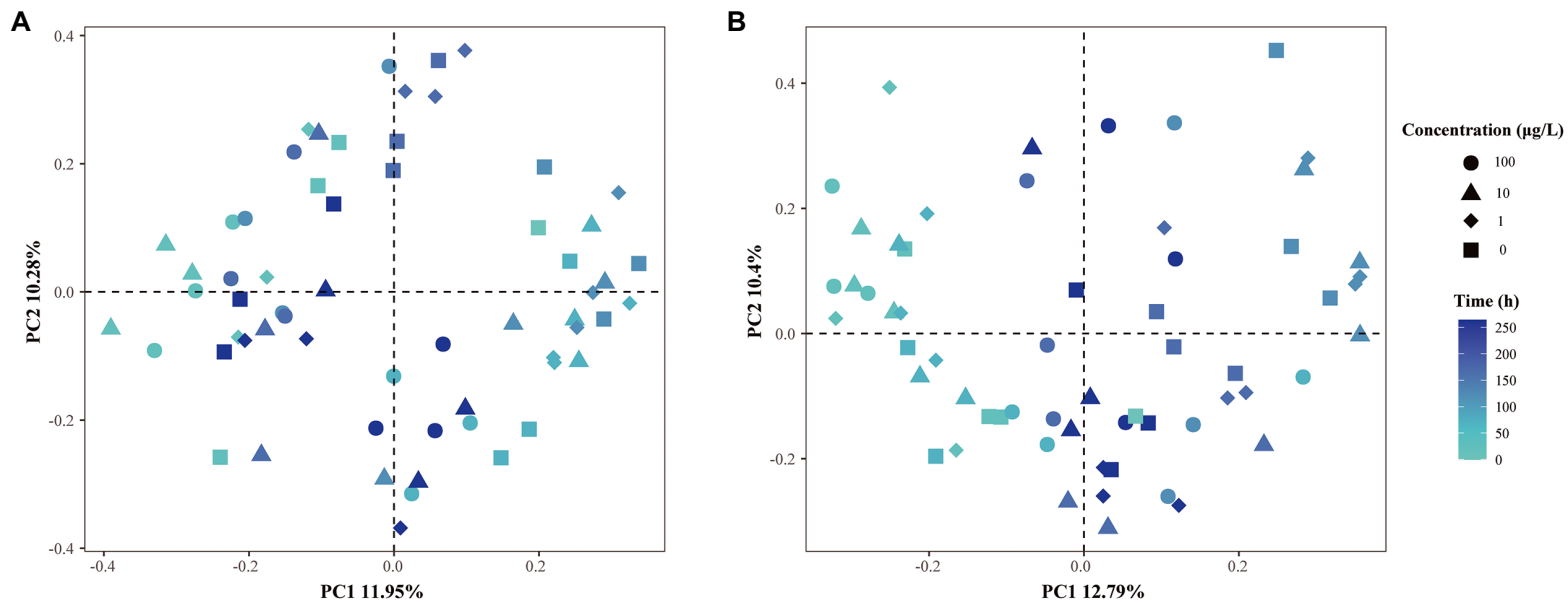
PCoA analysis of β-dispersion based on different grouping principles. **(A)** Distances between samples from site N2 with different concentrations of E1 added at different time scale; **(B)** distances between samples from site N15 with different concentrations of E1 added at different time scale. Different shapes represent different concentrations of E1. The color of dots from light to dark represents changes over time.

β-diversity dispersion measures were applied in the microbial community structure studies to show the destabilization of bacterial opportunism in stressed environments (Zhao et al., 2017). Hence, an PCoA analysis plot based on Bray–Curtis dissimilarity was performed to investigate the shift in β-diversity of the microbial communities (Figure 3), and ANOSIM was used to determine the difference between the various groups. As shown in Figure 3, we did not observe clear separations when the grouping was based on exposure concentrations, where different shapes represent different concentrations of E1. The degradation curves of E1 at different concentrations show that E1 was rapidly degraded in the *in-situ* water samples for 12–24 h when the added E1 concentrations were 1 μg/l and 10 μg/l, and the sampling time for the microbial community structure analysis was 0, 24, 72, 120, 168 and 264 h. Thus there would be no continuous E1 pressure at the next three or four sampling time points on the community structure of bacteria. Therefore, we could focus on the sampling time to explore how the community structures were disturbed by E1 with different concentrations overtime and analyze whether there were significant inter-group differences. The results show the dissimilarities in corresponding microbial communities were slightly more significant than the differences in concentrations. This suggests the community compositions of the water samples from sites N2 and N15 were affected by both sampling time and E1 concentrations. After 1 μg/l and 10 μg/l E1 were degraded, the evolution of bacterial community structure would change compared to the samples exposed to 100 μg/l E1. The above results indicate that both time and E1 concentrations shaped the structure of the microbial communities.

### Microbial community composition under different treatment conditions

**Figure 4:**
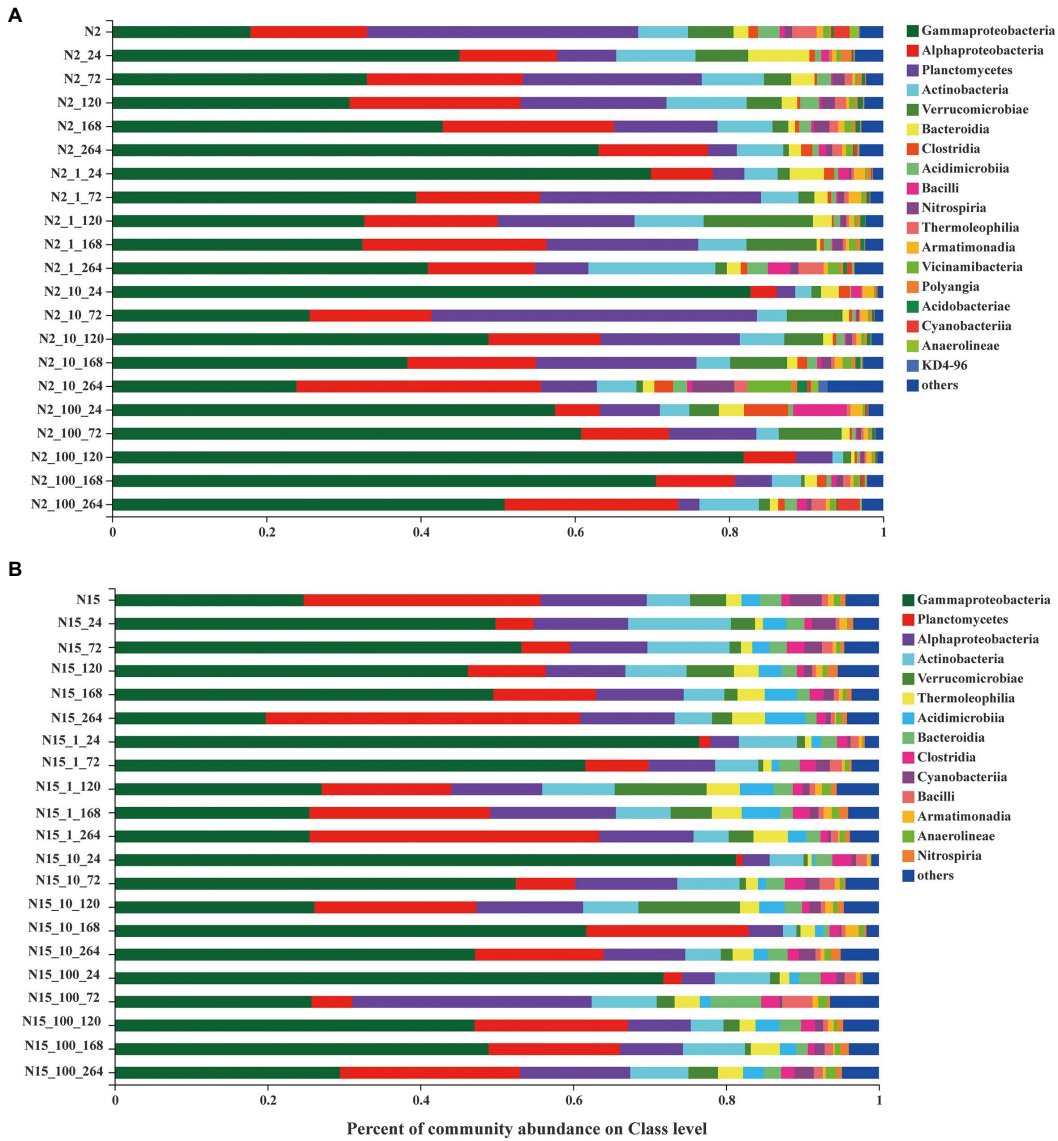
Bacterial community composition of different concentrations treatment groups on the Class level. **(A)** site N2; **(B)** site N15. (Each color represents one taxon across all samples. Less abundant taxa <1% are classified into the others). The last digit of the ordinate marker represents the sampling time.

The bacterial communities of all the treatment groups with different concentrations of E1 and sampling time were examined at the class level based on the mean OTUs corresponding to the three replicates (Figure 4). Overall, with the increase of exogenous E1 concentrations, the number of OTUs decreased in both N2_100 and N15_100. The dominant bacteria classes were *Gammaproteobacteria*, *Alphaproteobacteria*, *Planctomycetes*, *Actinobacteria* and *Verrucomicrobiase* within each group. *Alphaproteobacteria* changed stochastically without E1 on the time scale, while in all the N2 groups with E1, *Alphaproteobacteria* were enriched with sampling time as a pattern of change under external pressure. The variation in the abundance of *Gammaproteobacteria* decreased with sampling time in all the N2 groups with E1, indicating that E1 was a negative factor for *Gammaproteobacteria*. The abundance of bacteria was slightly different in the treatment groups of sample N15. The dominant bacteria classes were *Gammaproteobacteria*, *Planctomycetes*, *Alphaproteobacteria*, *Actinobacteria* and *Verrucomicrobiase*. *Planctomycetes* were enriched in the groups of N15 with E1. The results show that when the two water samples were exposed to E1 under the same concentration gradient, there was a difference in the bacterial community structure.

### Deterministic processes dominated the bacterial assembly based on *R*^2^ values

**Figure 5:**
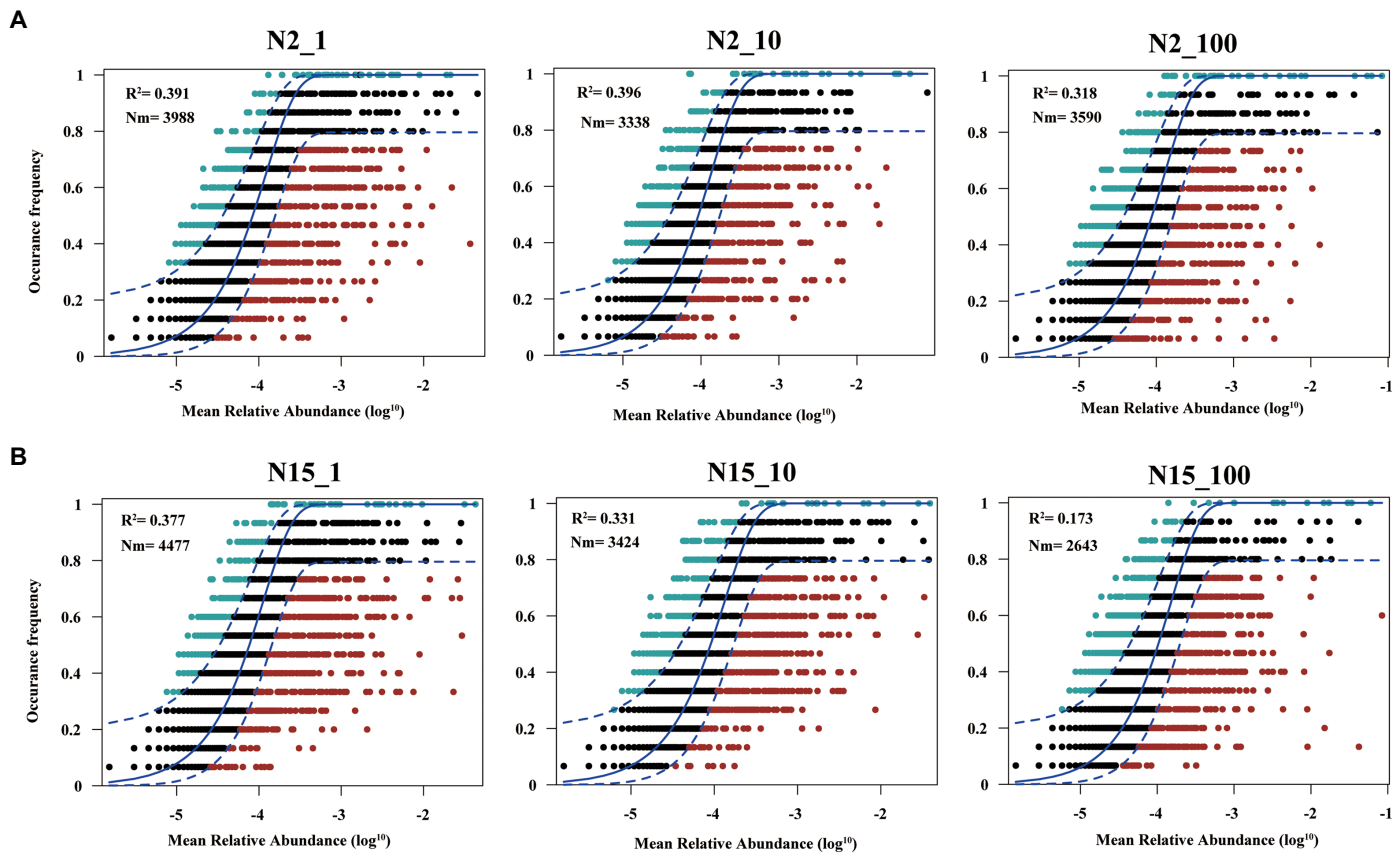
Fit of the neutral model to assess the effects of random dispersal and ecological drift on the assembly of bacteria communities. **(A)** Sample N2 with 1 μg/l, 10 μg/l and 100 μg/l E1 (labeled as N2_1, N2_10 and N2_100, respectively); **(B)** Sample N15 with 1 μg/l, 10 μg/l and 100 μg/l E1 (labeled as N15_1, N15_10, and N15_100, respectively). OTUs that occur more frequently than predicted by the model are shown in green while those that occur less frequently than predicted are shown in red. Dashed lines represent 95% confidence intervals around the model prediction (blue line).

Overall, the frequency of OTUs occurred in different datasets showed a moderate fit to the neutral model (Figure 5; Supplementary Figure S1). However, the fit of the model varied among different treatment groups of the two water samples. In sample N15 (Figure 5B), the value of R^2^ was in an order of: N15_1 (*R*^2^ = 0.377) > N15_10 (*R*^2^ = 0.331) > N15_100 (*R*^2^ = 0.173), which indicated that higher concentrations of E1 leaded to the diffusion restriction during the community assembly process. As shown in Supplementary Figure S1, *R*^2^ in the N15 sample without E1 was 0.408. Although it meant the deterministic dominated in the community assembly process, the diffusion restriction was smaller compared with the treatment group with a high concentration of E1. Also in sample N2 (Figure 5A; Supplementary Figure S1), the diffusion restriction of microbial community in the treatment group supplemented with 100 ug/l E1 was greater than those supplemented with low concentrations of E1 or without E1.

## Discussion

The structure and function of microbial communities are strongly affected by environmental contaminants. Therefore, a better understanding of the effects of these environmental contaminants on the associated microbial communities is crucial in river microbial ecology (Ahmed et al., 2019). We investigated the effects of E1 on microbial community dynamics in the river microcosm to understand the community assembly mechanisms. The results show that E1 could be degraded at low concentrations, e.g., 1 μg/l and 10 μg/l but was challenging to degrade at high concentrations, e.g., 100 μg/l in both water samples, implying that E1 with the concentration of 1 μg/l and 10 μg/l elicited a subsidy response. In comparison, E1 with the concentration of 100 μg/l elicited a stress response on microorganisms. The result differs from that of previous study using activated sludge to rapidly degrade high concentrations of estrogen (Chen et al., 2018). This disparity could be due to the *in-situ* microbial diversity and quantity of microorganisms in the microcosm. Still, our finding highlights that exploring the degradation characteristics of micropollutants with different concentrations are essential to understand the biodegradation potential by microorganisms. The degradation rates of estrogen in this study did not necessarily reflect the *in-situ* river water removal efficiency, because we have pretreated the water sample with a 0.4-μm membrane filter, which rules out the microorganisms larger than 0.4 μm, such as the algae. Also, we can not exclude the labile carbon sources’ influence in microbial capacity on degrading estrogen, although in this study, the starting concentration of the labile carbon sources in different groups of the same water sample should be identical. In addition, the result of the kill control experiment shows that the removal of estrogen is negligible without microorganisms and light.

We created an ideal environment to investigate the degradation of estrogen by spiking different concentrations of E1 to the same batch of water without interference from other environmental factors. To understand whether the degradation of estrogen altered the microorganisms’ community composition, we further analyzed the dynamic changes of microbial community structure before and after estrogen degradation at different concentrations of E1, especially for 1 μg/l and 10 μg/l, since when the concentration was 100 μg/l, estrogens were not degraded completely during the sampling period. We also used multiple methods of analysis to verify the discrepancy between different treatments under the varying concentrations of E1. The results showed that a high concentration of E1 resulted in a lower number of OTUs in the samples. In addition, under different spiking concentrations of E1, the difference in microbial community diversity among them was not noticeable, except the difference between the treatment group with 100 μg/L and the control group without E1 (Figure 2). β-diversity analysis of the 16S rRNA genes showed that both time and E1 concentrations shaped the diversity of microorganisms (Figure 3). Compared to the microorganisms which were not stimulated by E1, the variation in microbial communities stimulated by low concentrations of E1 was noticeable. We also considered the time scale of E1 effects on community assembly because the concentration of E1 was time-dependent. After low concentrations of E1(1 μg/l and 10 μg/l) were completely degraded, the interference effect on microbial community structure still existed. We also analyzed the bacterial community composition in all treatment groups at different times (Figure 4). The results showed that several classes of bacteria varied dynamically but remained dominant in different treatment groups, including *Gammaproteobacteria* and *Alphaproteobacteria*. E1 elicited a subsidy to the bacteria belonging to these classes, which could be supported by previous research regarding the bacterial degradation of estrogen (Balcom et al., 2016; Mainetti et al., 2021; Vilela et al., 2021). The variation of bacterial community structure in water samples from different locations may be mainly due to the composition of the bacterial community structure in the *in-situ* water sample (Ji et al., 2017).

To explore the difference in microbial community building mechanisms in different treatment groups, we used the neutral community model to explain whether deterministic or stochastic processes dominated on community assembly when disturbed by different concentrations of E1. Previous research has raised a hypothesis that if a microbial community was disturbed by micropollutants, the degradation of micropollutants altered the microbial community (Hu et al., 2017; Izabel-Shen et al., 2022). Both sensitive and tolerant taxa affected this process directly or indirectly (Freilich et al., 2011; Gao et al., 2019), and the microbial assembly mechanism is different at the beginning and end when exposed to micropollutants (Wolff et al., 2018). However, previous studies compared different kinds of micropollutants (Izabel-Shen et al., 2022). While in our study, we compared the community assembly mechanism in groups treated by different concentrations of E1 and also we collected samples continuously for 11 days even after E1 has been completely degraded, to explore the influence of micropollutants with different concentrations and whether it could last for a longer time even after the micropollutants have been removed. The results showed deterministic process was dominant in all the treatment groups throughout the sampling time, indicating that the addition of estrogen is a strong selection pressure for the microbial community. Overall, estimated migration rates tended to be lower in the high concentration of E1 treatment group than the low concentration of E1 treatment group, suggesting that communities became increasingly dispersal limited with the increasing concentrations of E1. This result is consistent with previous studies of other micropollutants (Wolff et al., 2018; Izabel-Shen et al., 2022) and reflects that the environmental effects of estrogen are persistent. A recent study also suggests estrogens as the priority pollution in surface water (Zhong et al., 2022), and their environmental occurrence should receive more attention.

In summary, we explored the response and assembly mechanisms of microbial community structure in water microcosm over time when exposed to different estrogen concentrations. Our results suggest that the microbial community structure could not be restored to the undisturbed state by estrogen even if disturbed by a low concentration of estrogen for a short time. Furthermore, we confirmed that endocrine disruptors, i.e., estrogens could cause long-term disturbance to the river water ecosystem. When we study the ecological effects of estrogen, we should also pay more attention to its biodegradation metabolites. In future work, we can identify the byproducts of estrogens and detect their changes to confirm the contribution of estrogens and the metabolites to the microbial community assembly.

## Data availability statement

The datasets presented in this study can be found in online repositories. The names of the repository/repositories and accession number(s) can be found at: https://www.ncbi.nlm.nih.gov/, PRJNA897809.

## Author contributions

DQ conceived and finished the study and performed the analyses, drew all pictures, and drafted the manuscript. YL and AH performed the research. NC contributed to sampling and water quality analysis. AH and C-PY framed the manuscript and contributed to revisions. All authors contributed to the article and approved the submitted version.

## Funding

This work was financially supported by the Fujian Province priority project (2020Y0086), the National Natural Science Foundation of China (41807411, 31870475, U1805244), the STS Project of Fujian-CAS (2021T3059, 2021T3014), and the FJIRSM and IUE Joint Research Fund (grant no.: RHZX-2019-005), the Scientific Research Foundation of Fujian University of Technology (GY-Z20083).

## Conflict of interest

The authors declare that the research was conducted in the absence of any commercial or financial relationships that could be construed as a potential conflict of interest.

## Publisher’s note

All claims expressed in this article are solely those of the authors and do not necessarily represent those of their affiliated organizations, or those of the publisher, the editors and the reviewers. Any product that may be evaluated in this article, or claim that may be made by its manufacturer, is not guaranteed or endorsed by the publisher.
