# Supplementary material for: Response and recovery mechanisms of river microorganisms to gradient concentrations of estrogen
Source: Front Microbiol. 2023 Feb 8;14:1109311. doi: 10.3389/fmicb.2023.1109311 (PMC9944024; doi:10.3389/fmicb.2023.1109311)
Supplement: Supplementary file 1 [file Data_Sheet_1.pdf]

Supplementary Material

1 Supplementary Tables

For more information on Supplementary Material and for details on the different file types accepted, please see here.Figures, tables, and images will be published under a Creative Commons CC-BY licence and permission must be obtained for use of copyrighted material from other sources (including re-published/adapted/modified/partial figures and images from the internet). It is the responsibility of the authors to acquire the licenses, to follow any citation instructions requested by third-party rights holders, and cover any supplementary charges.

Table S1: Sample information including location, temperature, electrical conductivity, pH, dissolved oxygen(DO), nitrite nitrogen(NO_2-N), nitrate nitrogen(NO_3-N), ammonia nitrogen(NH_4-N) The water depth is 0.5 meter.

Site No.	Longitude (°)	Latitude (°)	Temperature (℃)	Conductivity (μS/cm)	pH	DO (mg/L)	DO (%)	NO_2-N (mg/L)	NO_3-N (mg/L)	NH_4-N (mg/L)
N2	117.0866	25.1714	25.0	298.0	7.302	7.52	95.2	0.05	1.3	0.2053
N15	117.7898	24.5144	28.3	100.9	7.122	7.76	101.1	0.02	1.2	0.1124

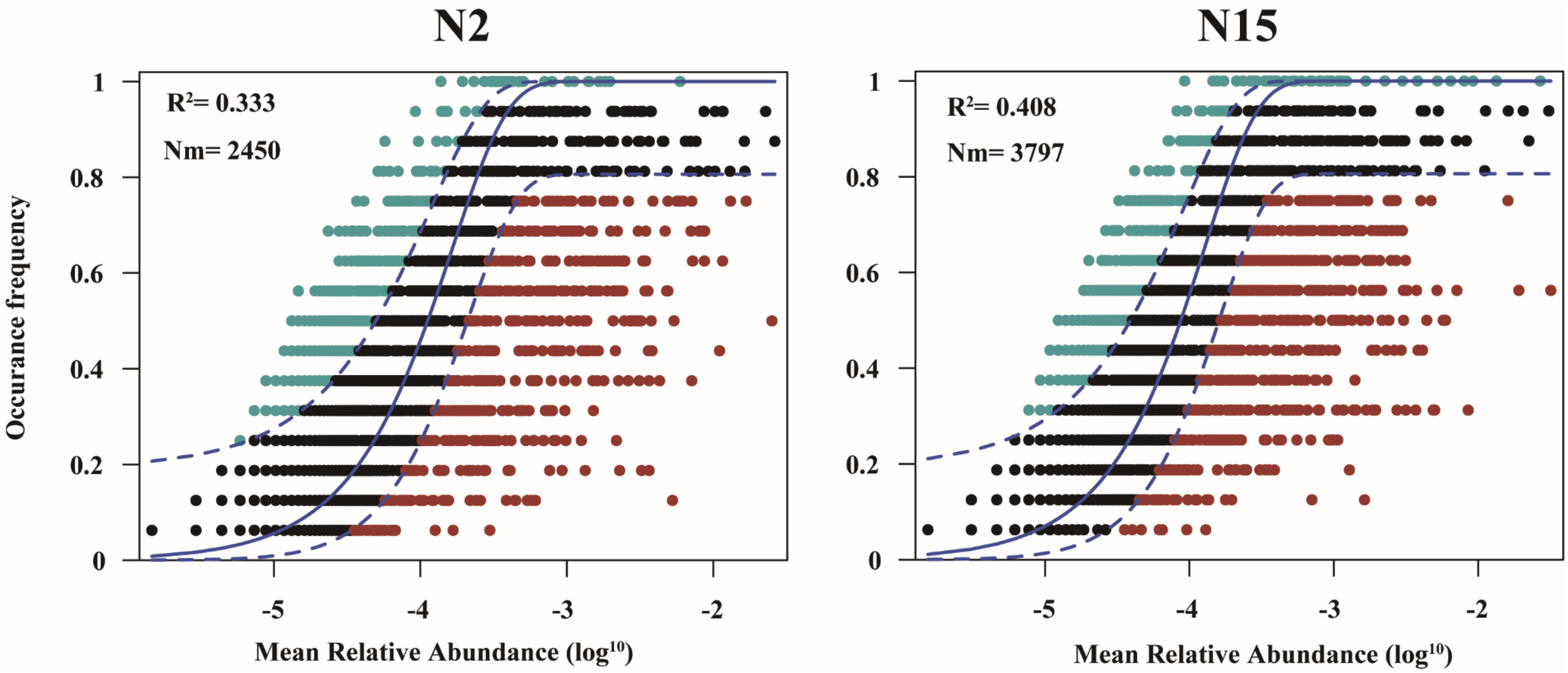

FIGURE S1 | Fit of the neutral model to assess the effects of random dispersal and ecological drift on the assembly of bacteria communities. Sample N2 and N15 Without E1. OTUs that occur more frequently than predicted by the model are shown in green while those that occur less frequently than predicted are shown in red. Dashed lines represent 95% confidence intervals around the model prediction (blue line).
